# Ergonomics appraisals in operating rooms

**DOI:** 10.1016/j.clinsp.2024.100439

**Published:** 2024-07-11

**Authors:** Jaime Solleiro Rodríguez, Juan Antonio Juanes Méndez, Fernando Blaya Haro

**Affiliations:** a Doctoral Programme Education in the Knowledge Society, University of Salamanca, Salamanca, Spain; b VisualMed Systems, University of Salamanca, Salamanca, Spain; c Analysis and Optical Characterization of Materials, Polytechnic University of Madrid, Madrid, Spain

**Keywords:** Occupational health, General surgery, Ergonomics, Risk, Work

## Abstract

This document presents the ergonomic assessments carried out by Spanish surgeons on the materials used within an operating room. With the objective of disseminating and raising awareness of the importance of ergonomics, this working group has compiled information from a previously conducted survey on musculoskeletal disorders associated with surgical work from the year 2022, obtaining feedback from 131 surgeons from 17 distinct specialties. A noteworthy 80.2 % of surveyed surgeons reported having experienced forced postures during surgery, and 96.9 % believe that their physical discomfort is a result of the posture adopted during operations. Such postures can result in the development of pathologies and may have a direct impact on work performance and even in extreme cases, it can lead to sick leave or early retirement. By providing their insights on electronic devices, surgical furniture, and instrumentation, surgeons can help identify areas for improvement in the practice of their profession.

## Highlights

• 100 % of the surgeons surveyed consider that improvements must be made in their work environment.
• Direct appraisals from surgeons about the main components in an operating room can be a valuable source of information for future developments in the field.
• Raise visibility in ergonomics, which may improve surgical conditions.

## Introduction

Ergonomics in the workplace has rightfully become a prominent topic within occupational diseases, especially in fields where repetitive, monotonous, or maintained tasks are common. This topic has been extensively discussed in the literature, and the common thread among all the fields studied is the need to emphasize that it is a current issue, typically complicated to address due to the numerous contributing factors. In many cases, it must be tailored to the job and the individual. Multiple studies have shown that musculoskeletal disorders related to work are a significant concern,^1^, ^2^, ^3^ and the field of surgery is one of the most affected.^4^^,^^5^ Throughout 2022, this working group conducted an exhaustive study of the ergonomic conditions experienced by surgeons and staff in an operating room through a national survey. The wealth of data collected demonstrates that professionals in this discipline continue to suffer from work-related musculoskeletal disorders, which can develop into chronic pathologies.

## Materials and methods

This observational study focuses on presenting the observations made by surgeons during a survey launched by this team to gather data on postural ergonomics. To this end, a national survey was launched, with respondents from 13 autonomous communities and 25 provinces of the Spanish territory. This is essential to understand the breadth and plurality of responses collected during the survey and the diversity of comments obtained since respondents have been from different fields of specialization and medical disciplines. The origin of the survey from which these data are extracted is based on other studies of this type and previous meta-analyses^6^, ^7^, ^8^, ^9^, ^10^ and has used similar questions to other survey models carried out^6^, ^7^, ^8^^,^^11^^,^^12^ with the exception that they have been reformulated and expanded to fit the topic and current reality, as well as focusing on the ergonomics of the workplace. Prior to its publication, the survey has been reviewed by expert surgeons who, after completing it, have suggested changes to make it more concise in some terms.

The survey is defined by 32 questions, divided into 5 sections and 9 sections:

Section 1: General data of the respondent.

Section 2: Data related to the surgical technique used.

Section 3: Posture during the operation.

Section 4: Common elements in an operating room, specifically:

• Operating table.
• Foot activation pedals.
• Instrumentation.
• Operating chair.
• Display devices.

Section 5: Free comments, where the respondent can provide or give information, they consider on the topic treated.

The collected data includes sociodemographic data, age, gender, and work-related data such as surgical specialty, operating hours, techniques used, working conditions, and ergonomics associated with the position. This work focuses on the Sections 4 and 5, on the free responses obtained in relation to the devices used in an operating room. This study has been designed according to STROBE guidelines.

### Tools

The survey form and the comments collected from surgeons were obtained through the use of Google Forms and processed using a spreadsheet compatible with Microsoft Excel. The data were analyzed using Statistical Package for Social Science for Windows version 25.0 (SPSS Inc.). The graphs were generated through cross-referencing with the same software.

### Sample

The information was sent to 304 public and private hospitals out of the 837 registered in Spain in 2020. This information was obtained from the Ministry of Health, Consumer Affairs and Social Welfare.

### Exclusions

The execution of this study was carried out by direct contact with the addresses provided by the Ministry of Health, Consumer Affairs and Social Welfare. Therefore, there is a possibility, albeit remote, that some emails may not have reached the intended recipients. In addition, hospitals without surgical practices and hospitals with less than 25 beds were excluded from the study. Out of the 304 hospitals contacted, evidence of having received the information was obtained from 196 of them, representing an incidence rate of 23.4 %.

### Data processing

The responses in free format have been collected and processed individually so that they have been compiled and generalized according to a criterion of similarity between them.

## Results

The responses of 131 surgeons to the questionnaire sent were collected, encompassing 17 different specialties including Cardiology, Dermatology, Digestive System, Breast Surgery, Maxillofacial Surgery, Orthopedic Surgery, Plastic Surgery, Thoracic Surgery, Vascular Surgery, Gynecological Surgery, Neurosurgery, Dentistry, Ophthalmology, Otolaryngology, Traumatology and Urology. This sample had a gender distribution of 46.9 % male, 52.3 % female, and 0.8 % who preferred not to disclose their gender. The age range of respondents was between 25 and 65 years old. The following Table 1 displays the interventions performed by these respondents in terms of the average time dedicated to surgeries, average duration of operations, and frequency of operations carried out throughout a week.

**Table 1:** Frequency and times related to surgery.

Weekly average in surgical interventions	
< 1-hour	Without results
Between 1 and 2 h	1.6 % (2)
Between 2 and 3 h	2.4 % (3)
Between 3 and 4 h	12.7 % (16)
> 4 h	83.3 % (105)


Average surgery duration	
< 1 hour	14.6 % (19)
Between 1 and 2 h	41.5 % (54)
Between 2 and 3 h	30.8 % (40)
Between 3 and 4 h	12.3 % (16)
> 4 horas	0.8 % (1)

Weekly frequency with which operations are carried out	
< 1 per week	3.2 % (4)
1 per week	11.9 % (15)
2 per week	40.5 % (51)
3 per week	16.7 % (21)
> 3 per week	27.8 % (35)

The data presented in this study allows for the easy identification of surgeons' involvement in these tasks as well as their weekly time dedication to them. It is evident that the vast majority of the sample, 83.3 %, dedicates more than 4 hours per week to being in the operating room, with a majority of surgeries lasting between 1 and 3 h, as observed in 72.3 % of cases.

**Fig. 1:**
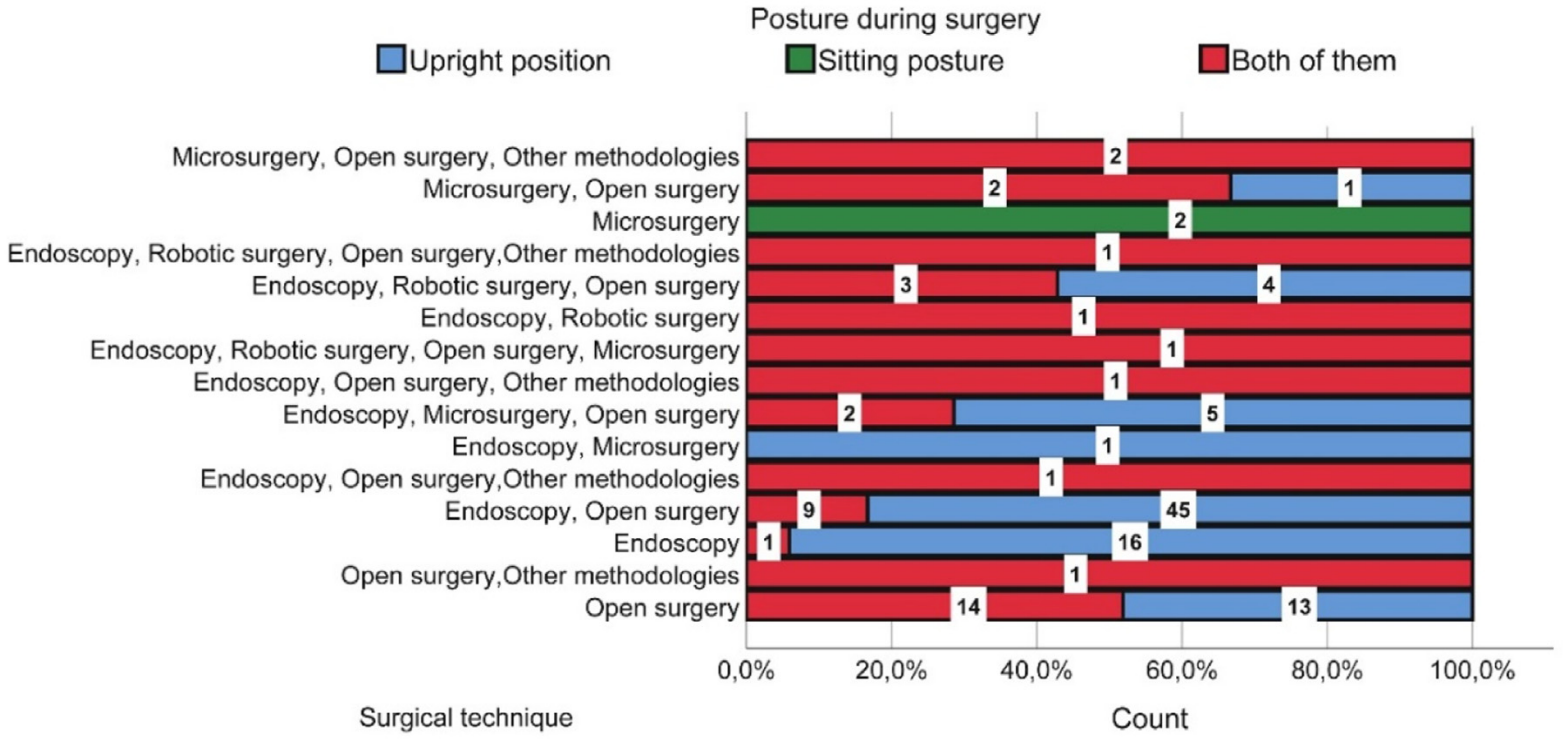
Surgical technique vs. Posture during surgery.

Regarding preferred operating positions, the following Fig. 1, collects the data categorized by surgical technique versus posture during surgery.

It can be observed that almost 65 % of the sample performs surgeries in an upright standing position. 97.6 % of the respondents stated that the position while operating is uncomfortable or painful. 79.4 % experience discomfort, fatigue, or some type of pain during surgery, with 48 % of them seeking medical attention to treat these discomforts, and only 8 % of them doing so on a regular basis. The most affected areas are the neck, where 31 % of the respondents report a high level of pain, the back with 43 % of the respondents, and 28 % report a high level of pain in the shoulders. Knowing these premises, surgeons have offered the following comments and improvements regarding the devices found in an operating room:

### Operating table

• The difference in height between surgeon and assistant can affect the operating table, its adaptation can be complex.
• There are comments referring to elevation range and setup options.
• Operating tables are not well adapted to sitting operations, and they can interfere with other devices.
• Problems for patients in surgical positions when under perineal and laparoscopic surgery have been reported, as well as in surgeries that require the patient to be seated.
• Leg supports and armrests should be improved to gain space in the operating area. Narrower headboards are also needed. All these elements should have better handling, in order to improve maneuverability.
• Protective covers for all items that are visible, such as screws, levers, etc.
• The inclusion of sterilized remote controls to move the table during surgery, if necessary, as well as the possibility of adding photophore lights to the operating area.

### Foot activation pedals

• The devices are not adapted to the size of the sanitary clogs and their soles are too soft, resulting in a loss of precision when used.
• Additionally, there have been comments suggesting that the pedals be replaced by keyboards or other visible elements close at hand. This is due to the fact that surgeons cannot see the pedals, which could lead to unintended activation.

### Instrumental

• There is an issue with the adaptation of instruments, as in some cases, the surgeon is not provided with the same material.
• A significant number of respondents have expressed interest in having more comfortable grips for the instrumentation.
• Improvements in the adaptability of the material to the surgeon's hand size is required.
• Improvement of ring handles rachets is also necessary.

### Operating chairs

As not all respondents regularly use operating chairs, the data has been extracted only from those who are familiar with their use. No remarkable opinions or comments have been received for the operating chair. The data obtained for operating chairs has been mixed, as shown in Table 2 below.

**Table 2:** Degree of well-being with operating chairs.

Degree of well-being in operating chairs during an intervention	
Very low degree of well-being	10.6 %
Low degree of well-being	24.3 %
Average degree of well-being	42.4 %
High degree of well-being	19.7 %
Very high degree of well-being	3 %

**Fig. 2:**
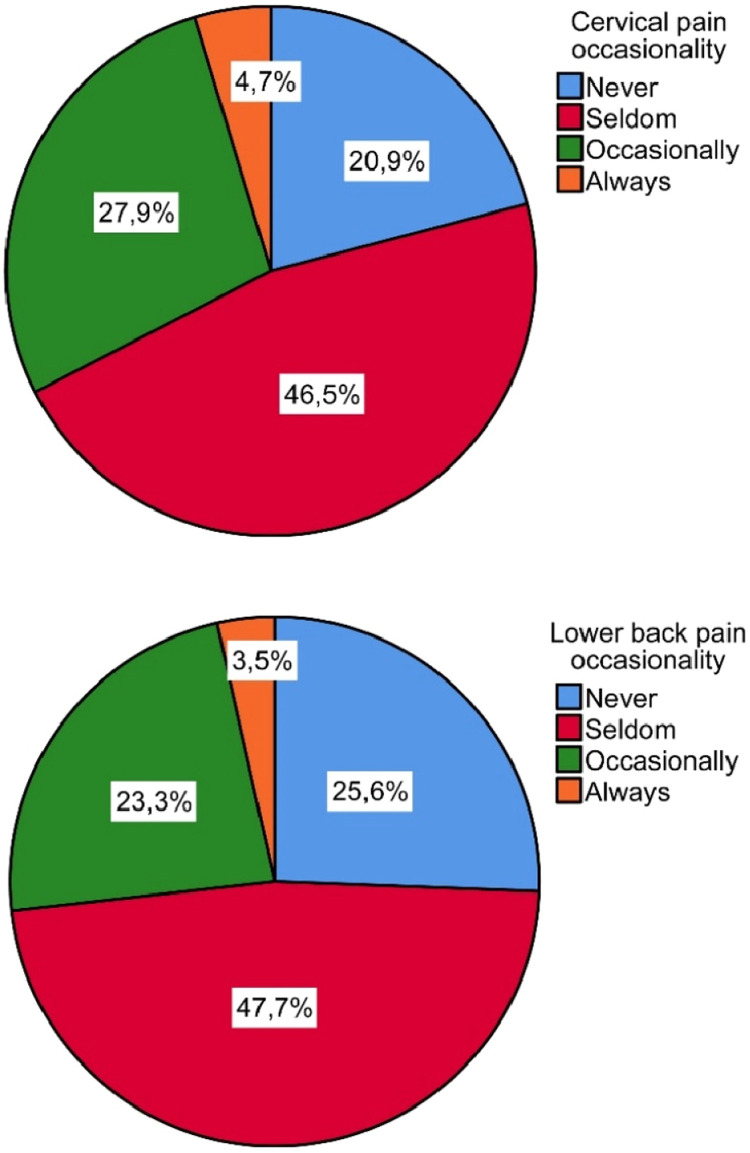
Occasionality of cervical pain and lower back pain with operating chairs.

The following graphic, Fig. 2, displays the disparity of these opinions concerning lower back and neck pain, which are the most frequently mentioned in the literature on the subject.

When asked about additional chair devices/accessories that support or rest some parts of the body, such as elbows or forearms during an intervention, 51.5 % of the sample group consider that they are useful, with 41.1 % of respondents saying they do not require them during an intervention, whilst only 7.8 % consider them expendable.

### Display devices

Similarly, to the previous case, both statistical results and free opinions are greatly influenced by the type of surgery and the conditions of the operation itself, obtaining homogeneous results. 34.7 % of the sample suffers from visual fatigue, while 38.7 % do not experience this problem However, 87.1 % consider that their performance can be affected by visual fatigue. The comments received for this type of element have been as follows:

• Improve the size and resolution of the devices to facilitate reading.
• Some of the received comments refer to the “ghosting effect”, which is related to the legibility of fixed letters due to dead pixels. This was common in screens that were on for many hours with fixed images, especially in older models, but it is rarely seen in new technologies, so it can be inferred that perhaps the equipment used by these respondents is outdated and requires replacement.
• The position of the monitors has also been widely discussed, with the possibility of changing them through a sterilized remote control or being able to adjust them using floating arms hanging from the ceiling. However, this type of technology cannot be installed in all operating rooms.

### Other comments

Other issues have been mentioned which are directly or indirectly related to job role ergonomics:

• Occasionally surgeons and their teams find themselves in temperatures as high as 35 degrees due to operating lights, thermal blankets, and waterproof gowns. Not working at a comfortable temperature can pose a risk.
• Although the surgeon's position can improve, the surgeon himself or his assistants generally have compromised or incorrect postures.

## Discussion

Despite the new techniques that are currently being investigated, and the improvements that are developed and applied in the field of patient treatment and care,^13^, ^14^, ^15^ there are still great differences in these same issues applied to health professionals. That is why, after obtaining the previous results, it can be said that:

It is common to find studies addressing the need to reach higher heights in some surgeries regarding the operating tables.^12^ Despite the board's knowledge of patient's positioning on the operating table and its procedures^16^^,^^17^ 80.2 % of the surveyed present doubts about table capacity. This absence of settings, specifically concerning the height of the patient, was previously defined as an element that causes fatigue.^6^ This issue was also gathered among surgeons’ opinions collected in this study. An element closely associated with operating tables is foot activation pedals; the outcomes obtained do not provide relevant data for improvement, although, it is true that its use could be substituted by a manual actuation device, so it is visible to the surgeon at all times, however, this is not always possible due to the type of surgery. Studies have shown adaptations carried out by the present study's surgeons.^8^

The improvements demanded by the surveyed in instrumentation, although they are unusual nowadays, can already be found on the market, and consequently, although their use is not widely extended, each day their implementation is closer. The influence of the angle use of instruments has also been studied, and the problems associated with it.^18^ The use of elements with less impact on ergonomics could increase ailments and develop associated pathologies, such as paraesthesia, arthrosis and epicondylitis. In the case of the data obtained for operating chairs, it shows 55 % of discomfort with the chair used, however, this element is highly dependent on the surgical use and the type of surgery. In any case, surgeons who use chairs suffer pain and discomfort in the neck, shoulders, and back. No differences have been found between operating chairs and desk chairs, although there are ergonomic differences in the areas of the neck and shoulders.^19^ Chairs with a larger supporting surface are preferred among surgeons, although the back and shoulder areas are still being affected besides the diverse types of chairs used.^20^

One of the most interesting points collected in this study is visual fatigue, given the maintaining focus on an element for a certain time, 61.3 % suffer pains of this kind, of which 34.7 % do regularly and 26.6 % occasionally. Remarks have been collected about “ghosting”, an anomaly of monitors produced by images overlapping,^20^ rarely common in new technologies. On the other hand, there is a relationship between display screens and work-related musculoskeletal disorders. Although no answers have been found in this regard, neck pain caused by posture fixation and wrong monitor positioning is very common, depending on the neck's flexion in each type of surgery. For example, its positioning in laparoscopy should be below the eye line.^21^

The comments gathered in this study refer to high temperatures for surgeons due to the clothes used. There are studies on air circulation within an OR addressing flow asymmetry regarding indoor ventilation conditions. This flow could be diverted due to the positioning of the lamps or monitors arranged close to the ceiling, causing it to affect surgeons.^22^ Other data has also been collected about dress wear causing fatigue/discomfort during surgery.^6^ This suggests that retraining, postural ergonomics, and Ambiental factors are as important as the other elements discussed in this work. Nevertheless, not enough data has been collected to consider this a major ergonomic issue. Additionally, important limitations are found in the study, specifically: the survey format implies answers being limited to a few options, which does not allow specific cases to be studied in depth. Similarly, another limitation is the number of respondents, despite efforts to obtain a greater number of results, the sample has not allowed obtaining more specific data on a specific specialty, as well as not being able to make more precise comparisons between different surgical typologies.

## Conclusions

This study presents contrasted information and opinions from expert surgeons on ergonomic criteria that directly affect their work in the operating room. Through this and other studies, the aim is to raise awareness about ergonomics and find the necessary avenues for their future resolution.

It can be said that there is a gap between the improvements and innovations demanded by professionals in the medical sector; in this case, focused on the surgical field, and the conditions and tools available in the market or workplaces. There are doubts about the operational capabilities of the operating tables, there is no consensus on the use of activation pedals, which suggests that new adaptations could be made to the current system. Additionally, the instruments and operating chairs, although they have a greater adaptation and availability in the market, do not reach the end user, so it could be said that there is also dissemination work to be done on these new technological developments. Finally, it is worth emphasizing the need to set up the operating rooms. As mentioned before, some problems are found with visualization as well as with air circulation or temperature control in some cases, therefore it is necessary to review and maintain facilities updated in order to improve the surgery team's performance during work.

Finally, it should be noted that 100 % of the respondents consider that improvements must be made in their work environment, and the appraisals about the main components presented in this document, can be a valuable source of information for future developments in the field. Moreover, sharing their opinions and preferences in a publication seeks to raise visibility on this issue, ergonomics, which is one of the major causes of work absenteeism in our territory.

## Conflicts of interest

The authors declare no conflicts of interest.
